# Supplementary material for: Nitrogen represses haustoria formation through abscisic acid in the parasitic plant Phtheirospermum japonicum
Source: Nat Commun. 2022 May 27;13:2976. doi: 10.1038/s41467-022-30550-x (PMC9142502; doi:10.1038/s41467-022-30550-x)
Supplement: Supplementary file 1 — Supplementary Information [file 41467_2022_30550_MOESM1_ESM.pdf]

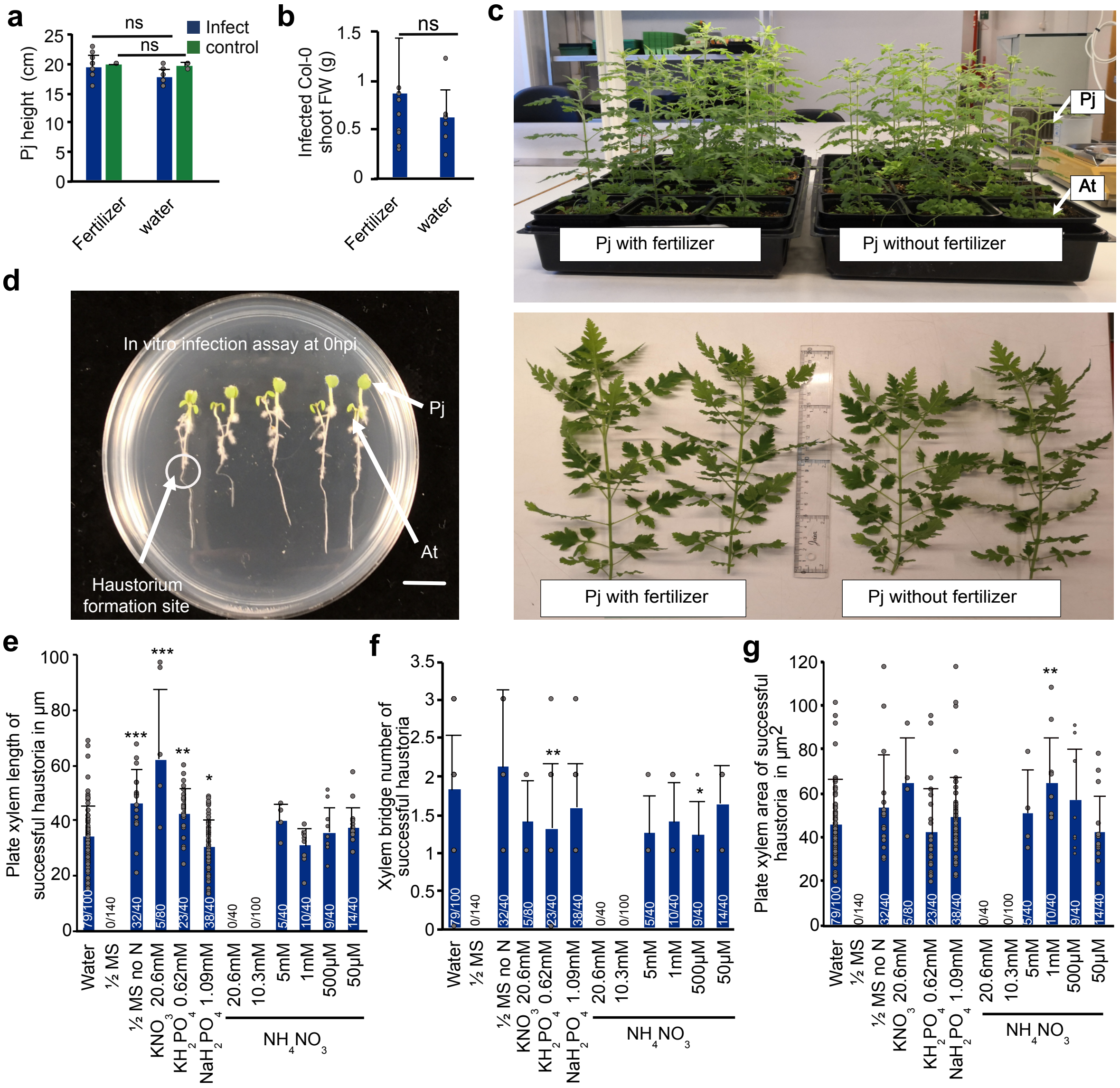

Supplementary Figure 1 Nutrient availability does not affect *P. japonicum* shoot growth and plate xylem size. (a-b) *P. japonicum* height and *Arabidopsis* Col-0 shoot fresh weight (FW) with or without fertilizer application during infection of *Arabidopsis* by *P. japonicum* (mean±SD, Fertilizer Pj infect n= 11 plants, Pj control n=3 plants, water infect n= 8 plants, control n= 4 plants, Col-0 fertilizer n= 11, water n= 8). (c) Photos of *P. japonicum* infecting with and without fertilizer application. (d) Photo of the *in vitro* infection assay set-up. Scale bar 1 cm. (e-g) Plate xylem length (µM), plate xylem area (µm^2) and xylem bridge number per haustorium under nutrient treatments (1/2MS, 1/2MS no N, KNO_3, NH_4NO_3, KH_2PO_4 or NaH_2PO_4), the numbers in the base of each column represent the number of measurements taken over the total number of plants, bars represent mean±SD. (a, b, e, f, g). Asterisks represent *P<0.05, **P<0.001, ***P<0.0001 compared to the water treatment, Student's t-test, two tailed. Source data provided.

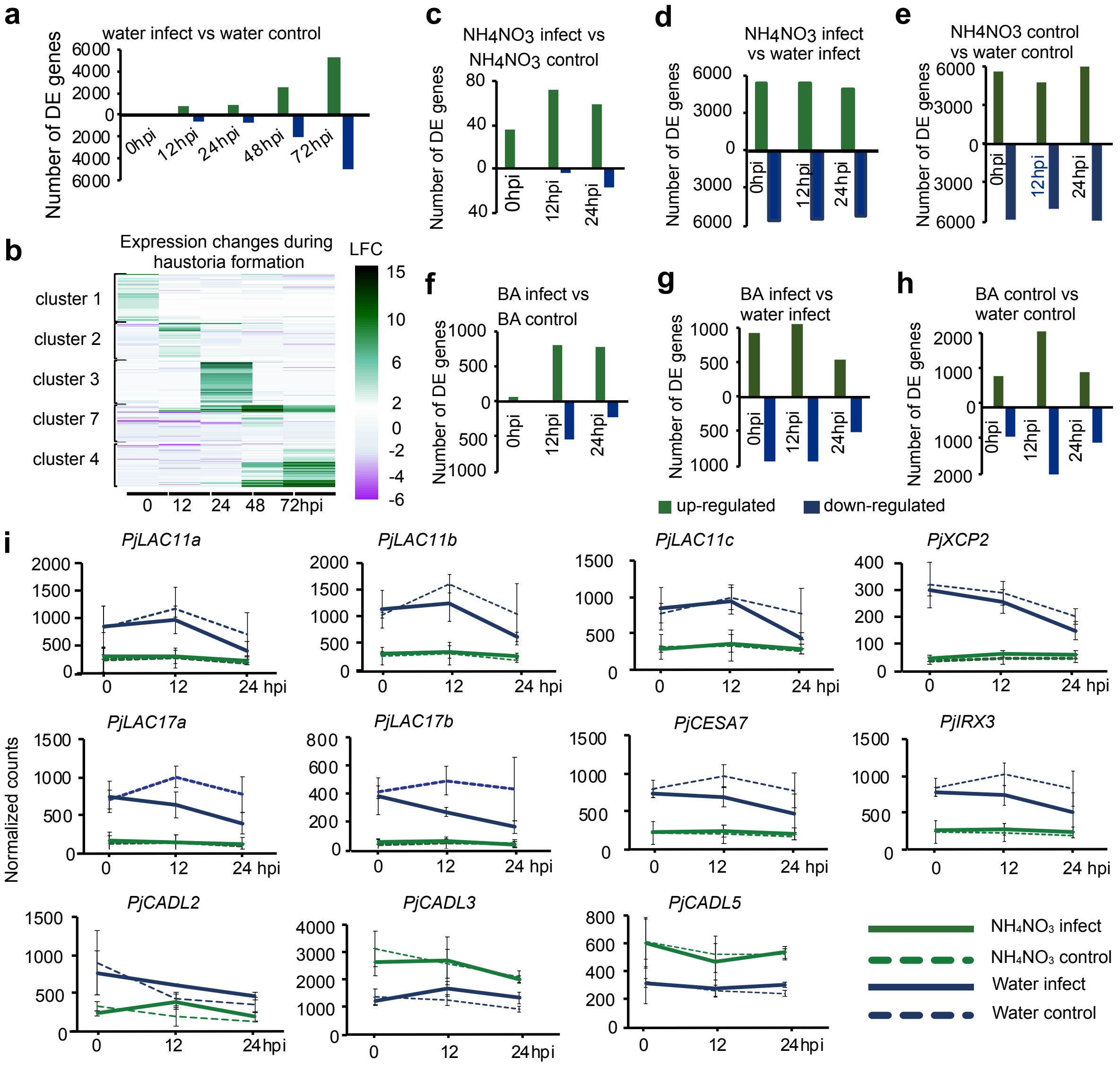

Supplementary Figure 2 NH_4NO_3 affects gene expression and xylem genes. (a, c-h) Number of genes differentially expressed over three or five time points in the water only, NH_4NO_3 and BA RNAseq dataset between *P. japonicum* infect and control and in the NH_4NO_3 or BA vs the water RNAseq dataset in *P. japonicum*. (b) Heatmap of the log2 fold change of gene subsets that belong to five co-expression clusters over five time points in the water-only RNAseq dataset between *P. japonicum* water infect vs water control. (i) Normalized counts of *PjXCP2, PjLAC11a,b,c, PjLAC17a,b, PjIRX3, PjCESA7, PjCADL*2, *PjCADL3*, *PjCADL5* over three time points shown for *P. japonicum* control and infect in the NH_4NO_3 and water treatment (mean±SD, n=3 libraries).

cluster 1		p-value	cluster2		p-value
GO:0016192	vesicle-mediated transport	3.10E-08	GO:0006260	DNA replication	2.50E-05
GO:0016567	protein ubiquitination	0.00023	GO:0006397	mRNA processing	0.00045
GO:0034613	cellular protein localization	0.00269	GO:0008654	phospholipid biosynthetic process	0.00178
GO:0023052	signalling	0.00093	GO:0006281	DNA repair	0.00047
GO:0015693	magnesium ion transport	0.01378	GO:0006413	translational initiation	0.00594
GO:0006808	regulation of nitrogen utilization	0.03685	GO:0009611	response to wounding	0.00841
			GO:1901657	glycosyl compound metabolic process	0.0073
			GO:0046039	GTP metabolic process	0.01748
			GO:0006396	RNA processing	1.60E-07
			GO:0006366	transcription by RNA polymerase II	0.03476
			GO:0030243	cellulose metabolic process	0.03904
**cluster3**		**p-value**	**cluster4**		**p-value**
GO:0007018	microtubule-based movement	3.80E-13	GO:0006508	proteolysis	6.70E-07
GO:0045944	positive regulation of transcription	2.90E-05	GO:2001141	regulation of RNA biosynthetic process	7.50E-05
GO:0000165	MAPK cascade	0.01294	GO:0005975	carbohydrate metabolic process	1.40E-06
GO:0033014	tetrapyrrole biosynthetic process	0.00602	GO:0005985	sucrose metabolic process	0.00490
GO:0007017	microtubule-based process	1.20E-12	GO:0010411	xyloglucan metabolic process	0.00570
GO:0044267	cellular protein metabolic process	0.00340	GO:0046274	lignin catabolic process	0.00710
			GO:0007064	mitotic sister chromatid cohesion	0.01870
			GO:0006952	defence response	0.03330
**cluster 5**		**p-value**	**cluster6**		**p-value**
GO:0055114	oxidation-reduction process	2.30E-07	GO:0016192	vesicle-mediated transport	3.30E-17
GO:0009060	aerobic respiration	3.80E-05	GO:0046907	intracellular transport	1.10E-11
GO:0006979	response to oxidative stress	0.0024	GO:0034613	cellular protein localization	1.70E-11
GO:0006561	proline biosynthetic process	0.0029	GO:0007264	small GTPase mediated signal transduction	1.80E-08
GO:0042744	hydrogen peroxide catabolic process	0.0178	GO:0030163	protein catabolic process	0.0001
GO:0009073	aromatic amino acid family biosynthetic process	0.0238	GO:0051225	spindle assembly	0.00017
GO:0030418	nicotianamine biosynthetic process	0.024	GO:0015991	ATP hydrolysis coupled proton transport	0.0005
GO:0045944	positive regulation of transcription	0.0281	GO:0072330	monocarboxylic acid biosynthetic process	0.00015
GO:0006633	fatty acid biosynthetic process	0.0293	GO:0016310	phosphorylation	0.0005
			GO:0015833	peptide transport	2.40E-11
			GO:0016052	carbohydrate catabolic process	0.01438
**cluster 7**		**p-value**	**cluster8**		**p-value**
GO:0055114	oxidation-reduction process	0.00022	GO:0006412	translation	< 1e-30
GO:0006779	porphyrin-containing compound biosynthesis	0.00046	GO:0022613	ribonucleoprotein complex biogenesis	7.80E-22
GO:0030001	metal ion transport	0.00027	GO:0006396	RNA processing	2.30E-17
GO:0006749	glutathione metabolic process	0.00666	GO:0034660	ncRNA metabolic process	2.50E-14
GO:0055085	transmembrane transport	0.00461	GO:0009451	RNA modification	8.70E-09
GO:0051252	regulation of RNA metabolic process	0.06845	GO:0006457	protein folding	1.10E-07
GO:0009690	cytokinin metabolic process	0.02798	GO:0032259	methylation	1.50E-08
GO:0006979	response to oxidative stress	0.02835	GO:0071826	ribonucleoprotein complex subunit	5.90E-08
			GO:0016071	mRNA metabolic process	2.40E-07
			GO:0042455	ribonucleoside biosynthetic process	1.00E-06
			GO:0009089	lysine biosynthetic process	3.60E-05

Supplementary Figure 3 Gene ontology of the co-expression clusters. Gene ontology analysis for the differentially expressed genes assigned to each co-expression cluster. GO categories with the lowest p-values are shown (P<0.05 Fisher's exact test). Source data provided.

a

NH_4NO_3 control vs water control up-regulated								
0hpi		p-value	12hpi		p-value	24hpi		p-value
GO:2000028	regulation of photoperiodism	0.0001	GO:0008272	sulfate transport	0.0006	GO:0009607	response to biotic stimulus	4.30E-07
GO:0051252	RNA metabolism regulation	0.0014	GO:0055114	oxidation-reduction process	0.0006	GO:0051252	RNA metabolism regulation	7.80E-06
GO:0006000	fructose metabolic process	0.0012	GO:0051252	RNA metabolism regulation	0.0040	GO:0055114	oxidation-reduction process	1.20E-04
GO:0005992	trehalose biosynthetic process	0.0021	GO:0005992	trehalose biosynthesis	0.0013	GO:0019344	cysteine biosynthetic process	0.0003
GO:0015698	inorganic anion transport	0.0006	GO:0009584	detection of visible light	0.0017	GO:0006952	defense response	0.0012
GO:0015969	guanosine tetraphosphate metabolism	0.0023	GO:0018298	protein-chromophore linkage	0.0017	GO:0006563	L-serine metabolic process	0.0013
GO:0055114	oxidation-reduction process	0.0035	GO:0009607	response to biotic stimulus	0.0040	GO:0019318	hexose metabolic process	7.20E-05
GO:0015995	chlorophyll biosynthesis	0.0086	GO:0006001	fructose catabolic process	0.0117	GO:0009785	blue light signaling pathway	0.0038
GO:0015979	photosynthesis	0.0028	GO:0034755	iron ion transmembrane transport	0.0117	GO:0006817	phosphate ion transport	0.0041
GO:0000160	phosphorelay signal transduction	0.0100	GO:0042128	nitrate assimilation	0.0117	GO:0015969	guanosine tetraphosphate metabolism	0.0041
NH_4NO_3 control vs water control down-regulated								
0hpi		p-value	12hpi		p-value	24hpi		p-value
GO:0042737	drug catabolic process	8.40E-06	GO:0046274	lignin catabolic process	5.60E-08	GO:0007017	microtubule-based process	1.30E-08
GO:0046274	lignin catabolic process	0.0001	GO:0042737	drug catabolic process	1.10E-06	GO:0046274	lignin catabolic process	9.10E-05
GO:0071554	cell wall organization/biogenesis	0.0001	GO:0055114	oxidation-reduction process	0.0009	GO:0008610	lipid biosynthetic process	6.20E-05
GO:0006857	oligopeptide transport	0.0031	GO:0006979	response to oxidative stress	0.0008	GO:0009690	cytokinin metabolic process	0.0011
GO:0006979	response to oxidative stress	0.0035	GO:0005975	carbohydrate metabolism	0.0003	GO:0006631	fatty acid metabolic process	0.0007
GO:0055085	transmembrane transport	0.0061	GO:0045492	xylan biosynthetic process	0.0057	GO:0009813	flavonoid biosynthesis	0.0026
GO:0005975	carbohydrate metabolism	0.0057	GO:0007017	microtubule-based process	0.0066	GO:0042737	drug catabolic process	0.0005
GO:0009269	response to desiccation	0.0269	GO:0000079	regulation of protein synthesis	0.0082	GO:0008202	steroid metabolic process	0.0040
GO:0044264	polysaccharide metabolism	0.0344	GO:0015743	malate transport	0.0086	GO:0055114	oxidation-reduction process	0.0040
GO:0042219	amino acid catabolism	0.0366	GO:0006542	glutamine biosynthesis	0.0139	GO:0006555	methionine metabolism	0.0084

BA control vs water control up-regulated								
0hpi		p-value	12hpi		p-value	24hpi		p-value
GO:0009690	cytokinin metabolic process	1.50E-08	GO:0006260	DNA replication	1.10E-26	GO:0006412	translation	6.40E-07
GO:0000160	phosphorelay signal transduction	8.60E-08	GO:0006412	translation	1.40E-26	GO:0009690	cytokinin metabolic process	6.60E-05
GO:0055114	oxidation-reduction process	2.10E-06	GO:0006457	protein folding	6.00E-16	GO:0006457	protein folding	1.30E-04
GO:0006006	glucose metabolic process	0.0001	GO:0007018	microtubule-based movement	3.3E-13	GO:0009152	purine ribonucleotide synthesis	0.0004
GO:0006855	drug transmembrane transport	0.0004	GO:0006265	DNA topological change	10E-05	GO:0009168	ribonucleoside monophosphate synth	0.0004
GO:0009607	response to biotic stimulus	0.0027	GO:0006310	DNA recombination	5.50E-06	GO:0009206	ribonucleoside triphosphate synthesis	0.0005
GO:0042737	drug catabolic process	0.0018	GO:0009690	cytokinin metabolic process	0.0001	GO:0006855	drug transmembrane transp.	0.0008
GO:0008272	sulfate transport	0.0101	GO:0009082	amino acid biosynthesis	0.0003	GO:0006979	response to oxidative stress	0.0009
GO:0009664	plant-type cell wall organization	0.0177	GO:0006281	DNA repair	0.0004	GO:0042744	hydrogen peroxide catabolism	0.0027
GO:0006979	response to oxidative stress	0.0200	GO:0065004	protein-DNA complex assembly	0.0014	GO:0009664	cell wall organization	0.0048
BA control vs water control down-regulated								
0hpi		p-value	12hpi		p-value	24hpi		p-value
GO:0019419	sulfate reduction	0.00076	GO:0055114	oxidation-reduction process	7.10E-05	GO:0006355	regulation of transcription	4.60E-04
GO:0071555	cell wall organization	0.00275	GO:0046274	lignin catabolic process	7.70E-05	GO:0006857	oligopeptide transport	5.90E-04
GO:0006857	oligopeptide transport	0.00332	GO:0019344	cysteine biosynthesis	0.0004	GO:0019419	sulfate reduction	9.60E-04
GO:0048544	recognition of pollen	0.00918	GO:0006468	protein phosphorylation	0.0002	GO:0005992	trehalose biosynthesis	0.0024
GO:0046274	lignin catabolic process	0.01246	GO:0009607	response to biotic stimulus	0.0002	GO:0019344	cysteine biosynthesis	0.0041
GO:0055085	transmembrane transport	0.05784	GO:0005992	trehalose biosynthesis	0.0009	GO:0055085	transmembrane transport	0.0205
GO:0015936	coenzyme A metabolism	0.01453	GO:0051179	localization	0.0033	GO:0048544	recognition of pollen	0.0147
GO:0006270	DNA replication initiation	0.02401	GO:0042737	drug catabolic process	0.0013	GO:0055114	oxidation-reduction process	0.0121
GO:0070588	Ca ion transmembrane transp.	0.02401	GO:0070588	Ca ion transmembrane transp.	0.0017	GO:0016567	protein ubiquitination	0.0286
GO:0055114	oxidation-reduction process	0.01327	GO:0008283	cell proliferation	0.0024	GO:0006558	L-phenylalanine metabolism	0.0299

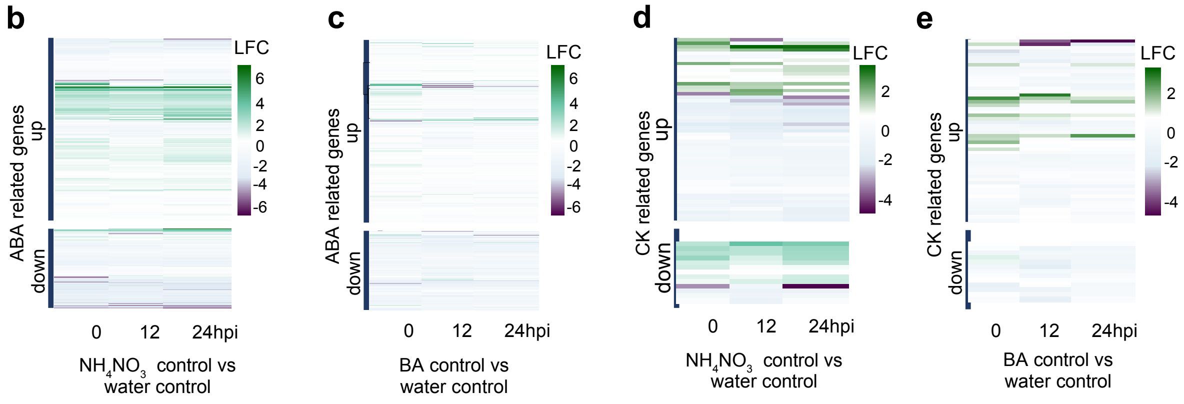

Supplementary Figure 4 Gene ontology analysis of the up and down regulated genes in NH_4NO_3 and BA control treatments. (a) Gene ontology analysis for genes differentially expressed during the three time points in the NH_4NO_3 control and BA control vs water control RNAseq datasets in *P. japonicum*, shown are the top 10 GO categories with P< 0.05 Fisher's exact test. (b-e) Heatmaps of the log2 fold change of 629 genes homologous to *Arabidopsis* ABA responsive genes (up or down regulated) and 67 genes homologous to *Arabidopsis* cytokinin responsive genes (up or down regulated) shown over three time points in the NH_4NO_3 control vs water control or in the BA control vs water control RNAseq datasets in *P. japonicum*.

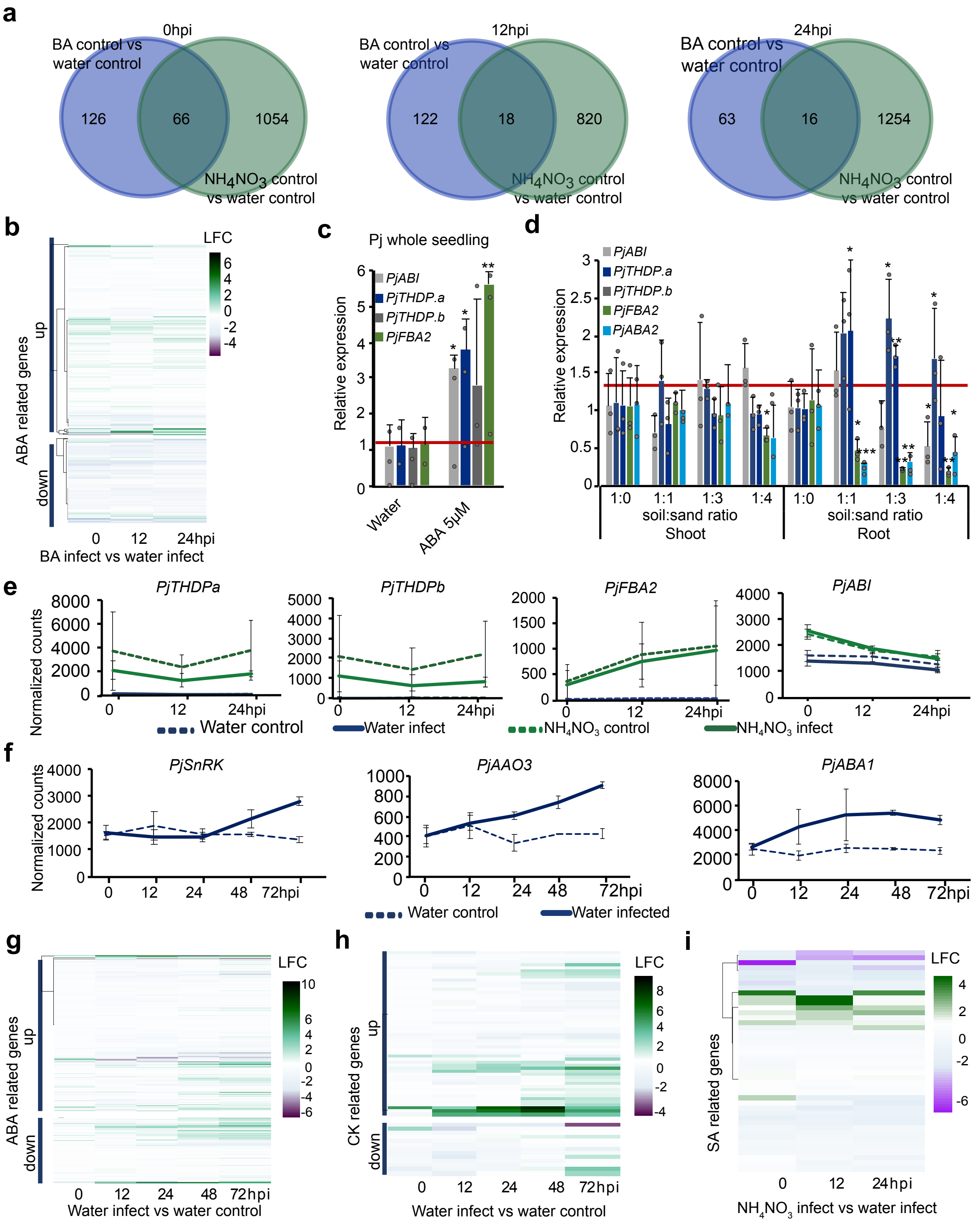

Supplementary Figure 5 Expression changes of ABA related genes. (a) Venn diagrams of the DE genes in BA and NH_4NO_3 treatments over three time points in *P. japonicum*. (b) Heatmap of the log2 fold change of 170 genes homologous to *Arabidopsis* ABA responsive genes shown over three time points in the BA infected vs water infected RNAseq dataset in *P. japonicum*. (c-d) Expression levels of *PjABI, PjFBA2, PjABA2 and PjTHDPa,b* with water, 5 μM ABA and various soil:sand ratios analyzed by RT-qPCR (mean±SD, n=4 plants per treatment, 3 replicates). (e-f) Normalized counts of *PjSnRK, PjAAO3, PjABA1, PjFBA2, PjTHDPa,b, PjABI* over five time points shown for *P. japonicum* water and NH_4NO_3 treatments (mean±SD, n=3 libraries). (g-h-i) Heatmaps of the log2 fold change of 629 genes homologous to *Arabidopsis* ABA responsive genes (up or down regulated), 67 genes homologous to *Arabidopsis* cytokinin responsive genes (up or down regulated) and 45 SA related genes shown over five time points in the water infect vs water control or over three time points in the NH_4NO_3 infect vs water infect RNAseq datasets in *P. japonicum.* (c, d)Asterisks represent *P<0.05, **P<0.001, ***P<0.0001 compared to control treatments, Student's t-test, two tailed. Source data provided.

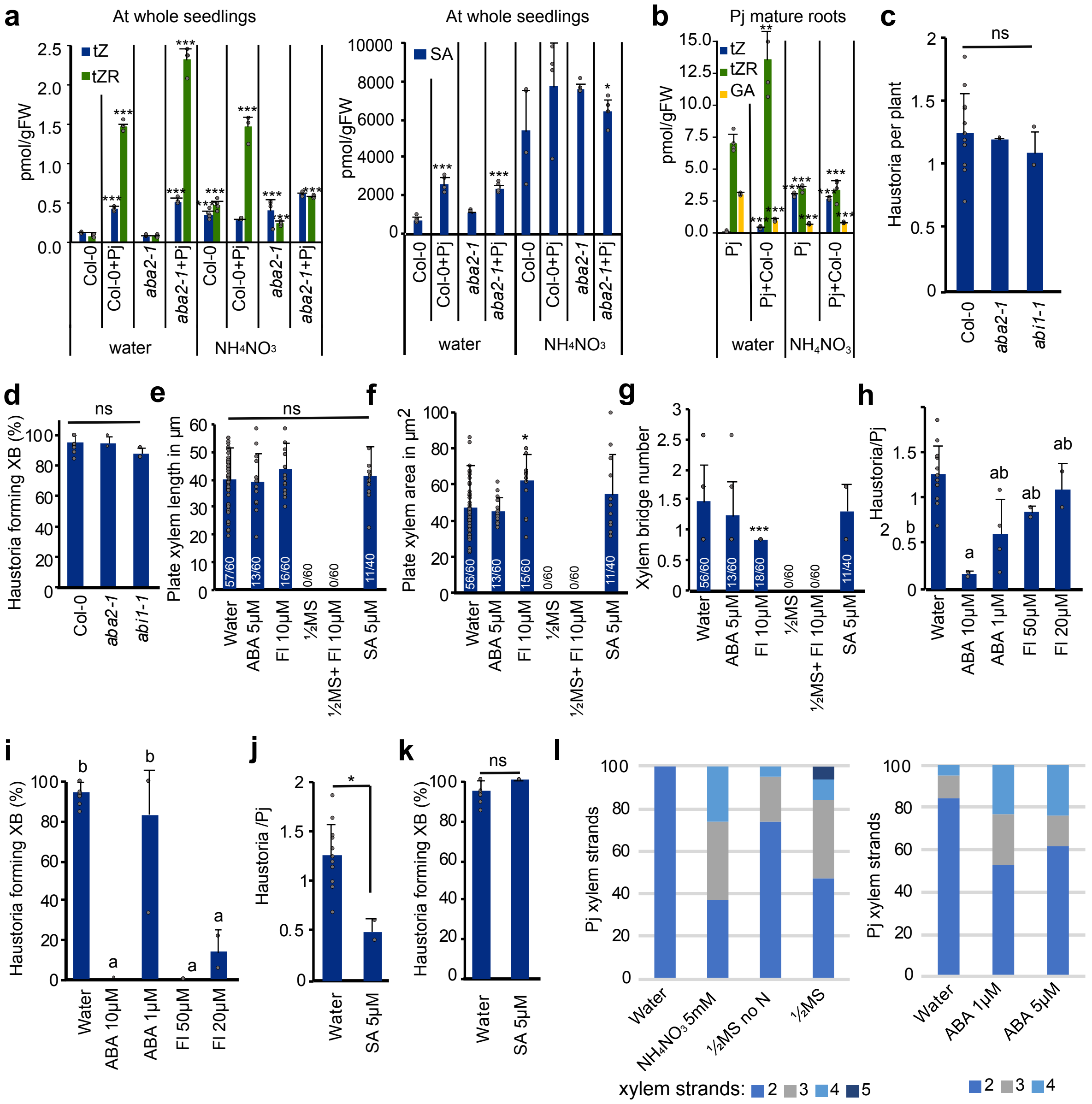

Supplementary Figure 6 Host ABA levels do not affect *P. japonicum* infection. (a-b) Hormonal quantification of salicylic acid (SA), gibberellic acid A1 (GA), trans-zeatin (tZ) and trans-zeatin riboside (tZR) in *Arabidopsis* (Col-0, *aba2*-1) whole seedlings and *P. japonicum* mature roots treated with 10.3 mM NH_4NO_3 (mean±SD, n= 4 plants per treatment, 4 replicates). (c-d) Average number of haustoria per *P. japonicum* seedling and xylem bridge formation percentage in *in vitro* infection assays with *Arabidopsis* Col-0, *aba2-1* and *abi1-1C* (*abi1-1*) as the host (mean±SD, n=20 plants per treatment, 3 replicates). (e-f-g) Plate xylem length (µM), plate xylem area (μm^2) and xylem bridge number per haustorium under ABA, fluridone, ½MS, ½MS + fluridone or SA treatments, the numbers in the base of each column represent the number of measurements taken over the total number of plants, bars represent mean±SD. (h-k) Average number of haustoria per *P. japonicum* seedling and xylem bridge formation percentage in *in vitro* infection assays with *Arabidopsis* Col-0 with ABA, fluridone, SA (mean±SD, n=20 plants per treatment per replicate, 2 replicates, ABA 1µM 4 replicates). (l) Number of lignified xylem strands at 2 mm from the root tip in *P. japonicum* seedlings treated with NH_4NO_3, ½MS, ½MS no N, ABA (n=19 roots per treatment). (c, d, h, i) Different letters represent one-way ANOVA followed by Tukey's HSD test P<0.05. (a, b, e, f, g, j, k) Asterisks represent *P<0.05, **P<0.001, ***P<0.0001 compared to Col-0 or water treatments, Student's t-test, two tailed. Source data provided.

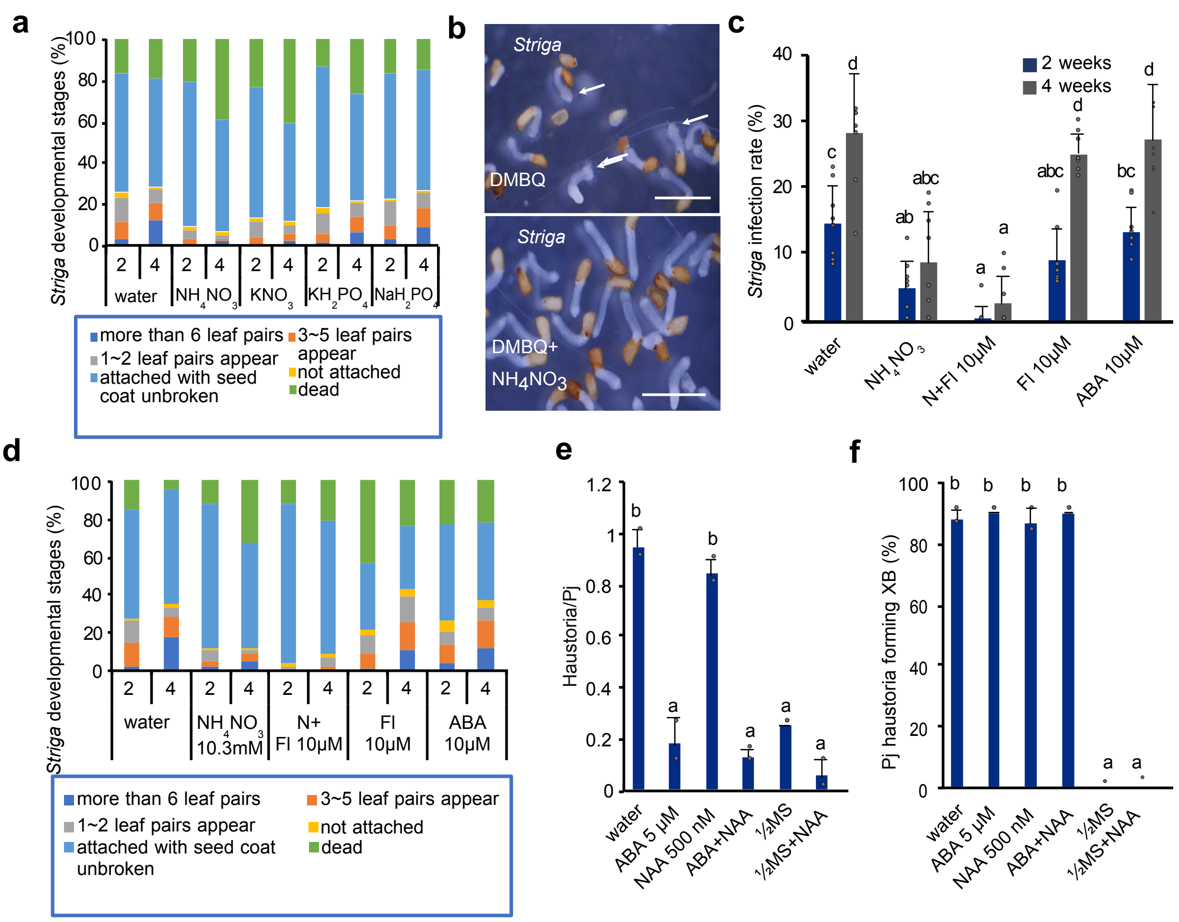

Supplementary Figure 7 Effect of ABA on *Striga*. (a) Effect of 20.6 mM KNO_3, 10.3 mM NH_4NO_3, 0.62 mM KH_2PO_4, 1.9 mM NaH_2PO_4 on *Striga* development at two and four weeks after infection (mean±SD, n=8 plants per treatment, 3 replicates). (b) Images of *Striga* haustorium formation assay with 1 µM DMBQ or 1 µM DMBQ+ 10.3 mM NH_4NO_3 at 1 day after treatment. The arrows denote pre-haustoria. Scale bars 1 mm. 3 replicates. (c) *Striga* infection rates (*Striga* with more than 3 leaves after rice infection over the total *Striga* number) at two and four weeks after infection with rice as a host under ABA, fluridone, 5 mM NH_4NO_3 or 5 mM NH_4NO_3 + fluridone treatments (mean±SD, n=8 plants per treatment, 3 replicates). (d) Effect of ABA, fluridone, NH_4NO_3 or NH_4NO_3 + fluridone treatments on *Striga* development at two and four weeks after infection (mean±SD, n=8 plants per treatment, 3 replicates). (e-f) Average number of haustoria per *P. japonicum* seedling and xylem bridge formation percentage in *in vitro* infection assays with *Arabidopsis* Col-0 with 5 µM ABA, 500 nM NAA, ½MS, 5 µM ABA+500 nM NAA and ½MS+500 nM NAA (mean±SD, n=20 plants per treatment, 2 replicates). (c, e, f) Different letters represent one-way ANOVA followed by Tukey's HSD test $P<0.05$. Source data provided.
