# Supplementary material for: Nitrogen represses haustoria formation through abscisic acid in the parasitic plant Phtheirospermum japonicum
Source: Nat Commun. 2022 May 27;13:2976. doi: 10.1038/s41467-022-30550-x (PMC9142502; doi:10.1038/s41467-022-30550-x)
Supplement: Supplementary file 3 — Description of Additional Supplementary Files [file 41467_2022_30550_MOESM3_ESM.pdf]

## Description of Additional Supplementary Files

File Name: Supplementary Data 1
Description: Gene lists in co-expression clusters

File Name: Supplementary Data 2
Description: Water infecting vs water not infecting Deseq2 results

File Name: Supplementary Data 3
Description: NH4NO3 infecting vs NH4NO3 not infecting Deseq2 results

File Name: Supplementary Data 4
Description: BA infecting vs BA not infecting Deseq2 results

File Name: Supplementary Data 5
Description: NH4NO3 infecting vs water infecting Deseq2 results

File Name: Supplementary Data 6
Description: NH4NO3not infecting vs water not infecting Deseq2 results

File Name: Supplementary Data 7
Description: BA infecting vs water infecting Deseq2 results

File Name: Supplementary Data 8
Description: Normalized counts of P. japonicum genes in all treatments and time points

File Name: Supplementary Data 9
Description: P. japonicum genes that are included in the heatmaps with the Arabidopsis gene they are homologous to

File Name: Supplementary Data 10
Description: List of primers

File Name: Supplementary Data 11
Description: Accession numbers of P. japonicum and S. hermonthica genes shown in figures
