# Supplementary material for: Nitrogen represses haustoria formation through abscisic acid in the parasitic plant Phtheirospermum japonicum
Source: Nat Commun. 2022 May 27;13:2976. doi: 10.1038/s41467-022-30550-x (PMC9142502; doi:10.1038/s41467-022-30550-x)
Supplement: Supplementary file 15 — Reporting Summary [file 41467_2022_30550_MOESM15_ESM.pdf]

Corresponding author(s): Charles W. Melnyk

Last updated by author(s): Apr 28, 2022

Reporting Summary

Nature Portfolio wishes to improve the reproducibility of the work that we publish. This form provides structure for consistency and transparency in reporting. For further information on Nature Portfolio policies, see our Editorial Policies and the Editorial Policy Checklist.

Statistics

For all statistical analyses, confirm that the following items are present in the figure legend, table legend, main text, or Methods section.

n/a	Confirmed	
☐	☒	The exact sample size (*n*) for each experimental group/condition, given as a discrete number and unit of measurement
☐	☒	A statement on whether measurements were taken from distinct samples or whether the same sample was measured repeatedly
☐	☒	The statistical test(s) used AND whether they are one- or two-sided<br>*Only common tests should be described solely by name; describe more complex techniques in the Methods section.*
☒	☐	A description of all covariates tested
☐	☒	A description of any assumptions or corrections, such as tests of normality and adjustment for multiple comparisons
☐	☒	A full description of the statistical parameters including central tendency (e.g. means) or other basic estimates (e.g. regression coefficient) AND variation (e.g. standard deviation) or associated estimates of uncertainty (e.g. confidence intervals)
☐	☒	For null hypothesis testing, the test statistic (e.g. *F*, *t*, *r*) with confidence intervals, effect sizes, degrees of freedom and *P* value noted<br>*Give P values as exact values whenever suitable.*
☒	☐	For Bayesian analysis, information on the choice of priors and Markov chain Monte Carlo settings
☒	☐	For hierarchical and complex designs, identification of the appropriate level for tests and full reporting of outcomes
☒	☐	Estimates of effect sizes (e.g. Cohen's *d*, Pearson's *r*), indicating how they were calculated

Our web collection on statistics for biologists contains articles on many of the points above.

Software and code

Policy information about availability of computer code

Data collection	no software was used
Data analysis	For the RNAseq data analysis presented in this manuscript we used the following published softwares: fastp 0.20.0, STAR 2.7.2b, FeatureCounts 1.6.4.p4, Deseq2 1.24.0, Mfuzz 2.44.0, InterProScan 5.3-69.0, topGO 2.36.0, tBLASTp 2.9.0, tBLASTn 2.9.0.

For manuscripts utilizing custom algorithms or software that are central to the research but not yet described in published literature, software must be made available to editors and reviewers. We strongly encourage code deposition in a community repository (e.g. GitHub). See the Nature Portfolio guidelines for submitting code & software for further information.

Data

Policy information about availability of data

All manuscripts must include a data availability statement. This statement should provide the following information, where applicable:

- Accession codes, unique identifiers, or web links for publicly available datasets
- A description of any restrictions on data availability
- For clinical datasets or third party data, please ensure that the statement adheres to our policy

The datasets generated during and/or analyzed during the current study are available in the Gene Expression Omnibus (http://www.ncbi.nlm.nih.gov/geo/) repository, under accession numbers GSE177484, or included in the article and supplementary files.

Field-specific reporting

Please select the one below that is the best fit for your research. If you are not sure, read the appropriate sections before making your selection.

☒ Life sciences ☐ Behavioural & social sciences ☐ Ecological, evolutionary & environmental sciences

For a reference copy of the document with all sections, see nature.com/documents/nr-reporting-summary-flat.pdf

Life sciences study design

All studies must disclose on these points even when the disclosure is negative.

Sample size	Sample size was 20 plants per sample for in vitro infection assays, haustorium induction assays and the various stainings. Sample size was 19 plants for the xylem strand assays. Sample size was 40 plants per sample for the RNAseq experiment. 4 plants per sample for the qPCR experiments. Sample size for hormonal quantification was 4 plants per sample. Sample size for Striga assays was 8 plants per sample. These sample sizes was determined after consulting the literature and testing for sufficient amount of material collection.
Data exclusions	no data were excluded
Replication	For in vitro infection assays, haustorium induction assays and the various stainings at least 2 biological replicates were prepared . For the rest experiments presented in this manuscript at least 3 biological replicates were prepared.(exact numbers of replicates are provided in the figure legends and Source data file).
Randomization	P. japonicum and S. hermonthica seedlings were randomly devided to control and treatment groups.
Blinding	This was not relevant to our study since the work described in the manuscript is on plants

Reporting for specific materials, systems and methods

We require information from authors about some types of materials, experimental systems and methods used in many studies. Here, indicate whether each material, system or method listed is relevant to your study. If you are not sure if a list item applies to your research, read the appropriate section before selecting a response.

Materials & experimental systems

n/a	Involved in the study	
☒	☐	Antibodies
☒	☐	Eukaryotic cell lines
☒	☐	Palaeontology and archaeology
☐	☒	Animals and other organisms
☒	☐	Human research participants
☒	☐	Clinical data
☒	☐	Dual use research of concern

Methods

n/a	Involved in the study	
☒	☐	ChIP-seq
☒	☐	Flow cytometry
☒	☐	MRI-based neuroimaging

Animals and other organisms

Policy information about studies involving animals; ARRIVE guidelines recommended for reporting animal research

Laboratory animals	the study did not involve laboratory animals
Wild animals	the study did not involve wild animals
Field-collected samples	the study did not involve field- collected samples
Ethics oversight	no ethical approval or guidance was required in this study since this did not apply to our work with the plants used in this manuscript (P. japonicum, S. hermonthica, A. thaliana and rice)

Note that full information on the approval of the study protocol must also be provided in the manuscript.
